# The Association between Adolescent's Weight Perception and Health Behaviors: Analysis of National Health and Nutrition Examination Survey Data, 2011–2014

**DOI:** 10.1155/2018/3547856

**Published:** 2018-04-23

**Authors:** Furong Xu, Mary L. Greaney, Steven A. Cohen, Deborah Riebe, Geoffrey W. Greene

**Affiliations:** ^1^Department of Kinesiology, University of Rhode Island, Independence Square II, Kingston, RI 02881, USA; ^2^Health Studies Program, University of Rhode Island, Independence Square II, Kingston, RI 02881, USA; ^3^Department of Nutrition and Food Sciences, University of Rhode Island, Fogarty Hall, Kingston, RI 02881, USA

## Abstract

The association between adolescents' weight perception and their physical activity (PA) and sedentary behaviors remains unclear. Therefore, these associations were explored using data from 2438 adolescents aged 12–19 years who participated in the National Health and Nutrition Examination 2011–2014 Survey. Respondents reported weight perception, and their weight perception accuracy was determined by examining whether the measured weight and perceived weight were concordant. Respondents also reported sedentary time (sitting time and screen time), PA, and intention to lose weight. Linear and logistic regression models were conducted to determine whether adolescents' PA, sedentary behaviors, and weight loss intention differed by weight perception and weight perception accuracy adjusted for demographic variables accounting for complex sampling. About one-quarter (21.4%) of the respondents had obesity. For respondents who perceived themselves as being overweight/fat, despite greater weight loss intention, males reported more sitting time (512.7 ± 16.3 versus 474.1 ± 10.2 minutes/day, *p* < 0.05) and females reported less PA (48.7 ± 5.0 versus 64.6 ± 3.3 minutes/day, *p* < 0.05) than respondents who perceived themselves as being normal weight. Similar patterns were observed for weight perception accuracy among individuals with obesity. Study results show that perceiving oneself as being overweight/fat regardless of accuracy was associated with more sedentary time for males or less PA for females despite higher weight loss intention.

## 1. Introduction

With 20.5% of adolescents aged 12–19 years in the United States (US) classified as being overweight or having obesity, excess weight is epidemic and a pressing public health challenge [1]. Obesity-related healthcare costs are increasing and will likely burden the US healthcare system in the future [2]. Adolescents with obesity are more likely to have obesity as adults and as a result be at increased risk for chronic diseases [3, 4]. Efforts to reduce adolescent obesity have had limited success [1]. Inadequate physical activity (PA) contributes to obesity: adolescents who are obese are less physically active and more sedentary than their normal weight counterparts [5]. The median self-reported minutes of daily moderate-to-vigorous physical activity (MVPA) for adolescents with obesity is lower than that for their nonoverweight counterparts (24.8 minutes/day versus 37.1 minutes/day) and is well below the recommended 60 minutes/day of MVPA [5]. Conversely, adolescents with obesity spent on average 43 minutes more per day sitting than their nonoverweight counterparts [5].

Weight perception, the way in which individuals view their weight, influences individuals' weight concerns [6, 7]. Although weight perception can be positive and may motivate weight control [8], negative weight perception can lead to extreme weight loss methods [6, 7]. Thus, weight perception should be considered when developing programs to combat obesity. The literature examining the association between adolescents' weight perception and their weight control strategies, especially PA, is inconsistent [6, 9–13]. Some of the existing research studies indicate that adolescents who view themselves as being overweight regardless of their actual weight status are more likely to intend to lose weight but less likely to be physically active than a perception of being normal weight [10, 13], while other research studies have found that perceiving oneself as overweight may be associated with more PA than perceiving oneself as being normal weight [9]. Furthermore, existing research findings are also inconsistent when examining PA among adolescents who accurately perceive themselves as being overweight or obese [6, 11, 13]. One study found stronger weight loss intention and more PA participation [11], while other studies have found a lack of PA and more negative weight control behaviors such as extreme dieting [6, 13]. Moreover, there is lack of research on adolescents' sitting time, in general. Given the emerging evidence of an association between sedentary behaviors and adolescent weight status [14], it is important to understand the association between weight perception and sedentary behaviors.

As rapid and diverse physical changes occur during adolescence and behavior patterns established in adolescence persist into adulthood [15], it is important to understand the relationships between weight perception and healthy behaviors to reduce obesity in adolescence [16, 17]. Yet, no identified studies that have examined weight perception and behaviors among US adolescents [6, 10–13] have assessed PA comprehensively or assessed sedentary sitting time in a nationally representative sample. Moreover, given the inconsistent evidence of the relationship between weight loss intention and healthy behaviors [11, 13], it is important to understand weight loss intention differences in relation to weight perception and weight perception accuracy. Accordingly, the purpose of this study was to examine the associations between adolescents' weight perception, weight perception accuracy, weight loss intention, PA, and sedentary behaviors, and how those potential associations may differ by sex. The primary hypothesis was that perceiving oneself as being overweight/fat would be associated with a greater intention to lose weight, more PA, and less sedentary time than those perceiving their weight as normal regardless of weight perception accuracy. The secondary hypothesis was that respondents who were accurately perceived themselves as having obesity would be more physically active, less sedentary, and have greater weight loss intentions than individuals with obesity who perceived their weight inaccurately.

## 2. Methods

The present study was a cross-sectional analysis of data from National Health and Nutrition Examination Survey (NHANES) 2011–2014. NHANES is a national health survey conducted by the Centers for Disease Control and Prevention (CDC) that includes questionnaires and an in-person examination conducted at a mobile examination center (MEC) [18]. The demographic questionnaire was completed by face-to-face interviews at the participants' homes by a parent or by the participants if 16 years of age or older. Participants aged 12–15 completed the PA questionnaire at an MEC while participants 16–19 years completed the questionnaire at home. Height and weight were measured at an MEC following standardized procedures [19].

### 2.1. Analytic Sample

The analytic sample was limited to adolescents who were 12–19 years old when they completed the NHANES examination (*n*=2636). Of the available respondents, 95.8% (*n*=2526) had complete data for all examined variables. Individuals who were underweight (*n*=88) based on calculated body mass index (BMI) were excluded due to the possibility that their weight status was related to psychological or physical pathology [20]. The final analytic sample included 2438 adolescents.

### 2.2. Measures

#### 2.2.1. Weight Perception

Weight perception was determined by participants' response to the question “how you consider your weight,” with the response options “overweight or fat,” “too thin,” and “about the right weight” [19]. Measured weight and height were used to calculate the BMI and to determine the weight status using CDC's sex-specific 2000 BMI-for-age growth reference for children and adolescents aged 2–20 years: underweight (BMI <5th percentile), normal weight (BMI 5th to <85th percentiles), overweight (BMI 85th to <95th percentiles), and obese (BMI ≥95th percentile) [21]. Weight perception accuracy (accurate versus inaccurate) was determined by comparing the perceived weight status and calculated the BMI weight status to determine if the two were concordant (accurate perceivers) or discordant (inaccurate perceivers).

#### 2.2.2. Weight Loss Intention

Weight loss intention was assessed by a single item that asked participants what they were trying to do about their weight, with response options: “lose weight,” “gain weight,” “stay the same,” or “do nothing.” Weight loss intention was recoded as “trying to lose weight” (yes versus no) [13, 22, 23]. Only respondents aged 12–15 years (*n*=1234) were asked this question [19].

#### 2.2.3. Physical Activity (PA)

PA was assessed using the Global Physical Activity Questionnaire (GPAQ) [19], a 16-item instrument assessing frequency and duration of PA participation in three domains: (1) work-related PA which was assessed by 6 items; (2) travel-related PA which was assessed by 3 items; and (3) leisure time PA which was assessed by 6 items [24]. GPAQ scores for each of the 3 domains were summed and used to calculate MVPA time expressed by daily MVPA minutes or weekly metabolic equivalent (MET) minutes [25]. Participants were categorized as to whether they met the World Health Organization PA recommendation of at least 1680 MET-minutes per week for children or adolescent 12–17 years of age, and 600 MET-minutes per week for individuals 18–19 years of age [25].

#### 2.2.4. Sedentary Time

Sedentary behaviors assessed in this study included sitting time and screen time. Sitting time was determined by participants' responses to a single item from the GPAQ that asked participants to report, in minutes, the time sitting or reclining on a typical day. Screen time was assessed by two items: (1) asked the participants to report the amount of time spent watching TV or videos and (2) asked the participants to report their daily computer use [25]. Responses (0–5+ hours per day) for both screen time items were summed and then used to determine whether participants met the American Academy of Pediatrics screen time guidelines (≤2 hours/day) [26].

#### 2.2.5. Covariates

Assessed demographic information included age, sex, race, parent education level (high school diploma or less versus some college or more), and the ratio of family income to poverty [19]. Responses for family income to poverty ratio (PIR) were used to create three household income categories: (1) PIR ≥3.5: at or above 350% of the poverty level; (2) 1.3 ≤ PIR < 3.5: between 130% and 350% of the poverty level; and (3) PIR <1.3: less than 130% of the poverty level [27].

### 2.3. Statistical Analysis

The MEC exam 2-year weights were used as the sample weight to conduct all analyses following the National Centre for Health Statistics recommendations [28]. Descriptive results were obtained for the sample and were presented as means ± standard error for continuous variables and frequencies and proportions for categorical variables. To assess the bivariate associations between pairs of key predictor and outcome variables and to determine whether PA or sedentary time varied by weight perception and weight perception accuracy in males and females, *p* values for continuous variables were obtained by performing PROC SURVEYREG (linear regression), and *p* values for categorical variables were obtained by performing PROC SURVEYLOGISTIC (linear logistic regression), with STRATA, CLUSTER, and WEIGHT statements in these two types of models, adjusted for age, race, PIR, and parental education level. PROC SURVEYFREQ (CHISQ, based on the Rao–Scott chi-square test with an adjusted *F* statistic) was also conducted to compare the difference in proportion when no adjustment was required.

Lastly, sensitivity analyses were conducted because of the difference in time spent on work-related PA between adolescents aged 16–19 and 12–15 years (42.7 ± 9.9 versus 11.0 ± 3.1 minutes/day, *p*=0.005) [29]. For this analysis, work-related PA was excluded from the total PA, and no meaningful differences in the results were found. Statistical significance was set at *p* < 0.05 for all analyses. All statistical analyses were conducted using SAS version 9.4 (SAS Institute Inc., Cary, NC).

## 3. Results

Of the 2438 adolescents in the study sample, approximately half (49.2%) were girls, 45.4% were racial/ethnic minorities, 42.9% had a parent who had a high school degree or less, and 33.7% of the participants lived below 130% of the poverty line (PIR <1.3). About one-fifth of boys (20.8%) and girls (21.9%) were classified as having obesity (See Table 1 for sample characteristics stratified by sex and weight status).

Sex-specific analysis revealed that PA, sitting time, and screen time differed by weight perception categories (thin, normal, and overweight/fat). For boys, individuals who considered themselves to be overweight/fat were more likely to intend to lose weight (87.5% versus 22.4%) than boys who perceived themselves as being normal weight; however, they were less likely to meet PA recommendations (64.0% versus 76.3%), spend more time sitting (512.7 ± 16.3 versus 474.1 ± 10.2 minutes/day), and had greater screen time (4.4 ± 0.2 versus 3.6 ± 0.1 hours/day) (Table 2). Among girls, individuals who perceived themselves as being overweight/fat were more likely to intend to lose weight (91.6% versus 29.4%) but reported less PA time (48.7 ± 5.0 versus 64.6 ± 3.3 minutes/day) than those who perceived themselves as normal weight (Table 2).

Comparisons of PA, sedentary time, and weight loss intentions among accurate weight perceivers revealed similar patterns as above (Table 3). In general, normal weight boys and girls perceived their weight accurately (87% and 87%, resp.) as did boys and girls who were obese (66% and 76%, resp.) but a smaller percentage of overweight boys and girls were accurate weight perceivers (21% and 49%, resp.). Boys with accurate weight perceptions who were obese were more likely to intend to lose weight than normal weight accurate perceivers (90.3% versus 12.8%); however, they were less likely to meet the PA (62.4% versus 77.4%) and screen time (24.7% versus 36.4%) recommendations and had more daily sitting time (535.6 ± 21.3 versus 468.1 ± 9.6 minutes/day) than normal weight boys. Among girls who perceived their weight status accurately, respondents with obesity were more likely to intend to lose weight (89.5% versus 19.8%) but were less likely to meet screen time recommendations (29.7% versus 42.9%) and were more physically inactive (41.0 ± 4.1 minutes/day versus 64.2 ± 3.5 minutes/day) than normal weight girls (Table 3).

Sex-specific analysis by weight perception accuracy among respondents classified as having obesity revealed differences in PA, sedentary time, and weight loss intentions by weight perception accuracy. Among boys who were obese, weight perception accuracy was associated with increased sitting time (535.6 ± 21.3 versus 497.8 ± 31.9 minutes/day, *p*=0.007), being less likely to meet screen time recommendations (24.7% versus 34.0%, *p*=0.036), and having a greater intention to lose weight (90.3% versus 56.4%, *p*=0.004) than inaccurate perceivers (Table 3). Among girls with obesity, accurate weight perceivers were less physically active (41.0 ± 4.1 versus 61.7 ± 12.2 minutes/day, *p*=0.005) and have a greater intention to lose weight (89.5% versus 66.9%, *p*=0.046) than those who inaccurately perceived their weight (Table 3).

## 4. Discussion

The current study examined adolescents' PA and sedentary behaviors by weight perception and weight perception accuracy in a nationally representative sample. We found that respondents, regardless of their weight perception accuracy, who perceived themselves as being overweight/fat were more sedentary or less physically active. This finding provides valuable insight into obesity intervention programs designed to help adolescents overcome barriers to PA participation and reduce sedentary time.

In total, 34% of boys and 24% of girls who were classified as obese perceived their weight status inaccurately, which is similar to the proportion (40% boys versus 23% girls) from the Youth Risk Behavior Surveillance System, 1999–2007 [11]. Results of the current study confirm prior research that has shown that both boys and girls who perceive their weight as overweight/fat are less physically active than boys and girls who perceive their weight as being normal, regardless of weight perception accuracy [11, 13]. This suggests that weight perception may be a determinant of PA participation for boys and girls. Moreover, study findings indicate that accurately perceiving oneself as being overweight/fat does not necessarily motivate individuals to engage in more PA, as there was no difference in PA among boys with obesity by weight perception accuracy. However, female respondents who accurately perceived themselves as being obese had less average daily PA time than inaccurate weight perceivers. This finding differs from that of Fan and Jin's who found that boys with accurate weight perception of overweight were less physically active, whereas there was no PA difference in girls [13]. These different findings could be due to sample differences as their samples were older (9–12th grade students), difference in actual weight categories (overweight and obese were combined), and differences in PA assessment [13, 19]. The current study assessed three types of PA (work, travel, and leisure) and is the first, to our knowledge, to explore weight perception and PA using multiple PA domains, which is important because adolescents' PA is not necessarily limited to recreational PA [30–32]. Around 40% of respondents in our study reported work- or travel-related PA. Thus, the current study adds to the literature as it provides evidence that PA occurs in different domains and suggests that adolescent obesity prevention program should be tailored to PA in different domains.

A novel study finding is that boys and not girls, who perceived themselves as being overweight/fat had greater sitting time in general and more screen time than boys who perceived themselves as being thin or normal weight. This finding of a specific difference in sitting time and screen time may help future health promotion efforts addressing sedentary behaviors. Moreover, when taking weight perception accuracy into consideration, both boys and girls were accurately perceived themselves as having obesity reported more screen time than those who accurately perceived themselves as being normal weight, which was consistent with that of Fan and Jin [13]. Since sitting time and screen time may be associated with an increased risk for obesity [33, 34], it is important to address sedentary behaviors via appropriate and effective guidance and support to reduce obesity among adolescents.

Results of this study also indicate that adolescents who viewed themselves as overweight/fat were more likely to express weight loss intentions than those who perceived themselves as being normal weight, which is consistent with previous research [13, 26], although weight loss intention was not associated with greater PA. Moreover, respondents with obesity who accurately perceived their weight status were more likely to express weight loss intention than inaccurate weight perceivers, which aligns with the findings of Fan and Jin [13]. This finding along with the study findings on PA and sedentary time indicates that adolescents who are aware of their weight and intend to lose weight may need appropriate and effective guidance and support to overcome barriers to PA and make desirable behavior changes. It is also important to recognize that weight loss intention can be associated with potentially dangerous weight control practices. Dieting is a common weight control practice among adolescents with obesity, although dieting is often counterproductive leading to long-term weight gain [35, 36]. However, assessing dieting behavior is beyond the scope of the present study. Increasing MVPA time and decreasing screen time are positive weight control strategies that contribute to a healthy lifestyle pattern [37]. Study findings suggest that weight perception is associated with weight loss intention; however, there is a need to address barriers to PA participation among adolescents perceiving themselves as overweight/fat to help them transform weight loss intentions into effective health promoting behaviors.

Study findings should be interpreted with caution due to the study's cross-sectional design, which does not allow for causality to be determined. In addition, PA, sitting time, and screen time were assessed by self-reported measures, which may induce bias. Despite these limitations, PA was assessed by a validated instrument that been widely used internationally and in similar studies of adolescent obesity [24, 38, 39]. Also, due to the secondary nature of this data analysis, we were limited to the questions already asked in the survey and not able to conduct more in-depth analyses. For example, the available response to the question assessing weight perception responses did not separate overweight and obese, and weight loss intention data were only available for participants aged 12–15 years.

Strengths of this study include the use of the recent nationally representative sample to investigate and the use of measured height and weight to calculate the BMI and to determine weight perception accuracy. In addition, the PA measure assessed multiple domains of PA, and no similar studies have included work and travel PA measures or sitting time. Lastly, a sensitivity analysis was conducted, which confirmed primary findings when work-related PA was excluded from the total PA time.

## 5. Conclusion

A perception of being overweight/fat in adolescents was associated with greater weight loss intention but more sedentary time for boys or less PA time for girls than a perception of being normal weight regardless of weight perception accuracy. Weight perception accuracy was not associated with more PA or less sedentary time among individuals with obesity despite their greater intention to lose weight. This finding is important for obesity prevention/intervention programs or future health promotion efforts aimed to help adolescents improve PA participation and reduce sedentary time.

## Figures and Tables

**Table 1:** Sample characteristics of adolescents aged 12–19 years in NHANES 2011–2014.

	Total	Boys				Girls			
		Normal	Overweight	Obese	*p* value	Normal	Overweight	Obese	*p* value
	*n*=2438	*n*=757 (62.4%)	*n*=210 (16.8%)	*n*=258 (20.8%)		*n*=749 (63.4%)	*n*=200 (14.7%)	*n*=264 (21.9%)	
Age (years)^$^	15.4 ± 0.1	15.5 ± 0.1	15.4 ± 0.3	15.4 ± 0.2	0.985	15.5 ± 0.1	15.7 ± 0.2	15.1 ± 0.2^c^	0.054
BMI (kg/m^2^)	24.3 ± 0.2	20.7 ± 0.1	25.6 ± 0.3^a^	32.9 ± 0.4^bc^	<0.001^∗^	20.6 ± 0.1	26.5 ± 0.2^a^	34.2 ± 0.5^bc^	<0.001^∗^
Race^&^, *n* (%)									
NH White	581 (54.6)	195 (55.8)	53 (53.4)	64 (51.0)	0.595	178 (58.5)	31 (40.9)^a^	60 (53.6)	0.017^∗^
NH Black	671 (14.7)	210 (14.8)	54 (14.1)	78 (15.6)	0.862	179 (12.4)	67 (21.0)^a^	83 (17.0)	0.007^∗^
Mexican American	512 (14.8)	141 (13.9)	52 (18.6)^a^	58 (17.2)	0.031^∗^	150 (13.0)	44 (16.1)	67 (16.4)	0.173
Others	674 (15.9)	211 (15.6)	51 (13.9)	58 (16.3)	0.81	242 (16.1)	58 (22.0)^a^	54 (13.0)^c^	0.049^∗^
Parent education^&^, *n* (%)									
High school or less	1190 (42.9)	336 (37.8)	105 (43.7)	145 (47.9)^b^	0.019^∗^	340 (40.9)	108 (52.7)^a^	156 (51.6)^b^	0.013^∗^
College or above	1166 (57.1)	385 (62.2)	99 (56.3)	110 (52.1)^b^	0.019^∗^	378 (59.1)	89 (47.3)^a^	105 (48.4)^b^	0.013^∗^
PIR^&^, *n* (%)									
PIR ≥3.5	480 (29.4)	176 (34.4)	40 (25.1)	42 (26.9)	0.214	171 (34.6)	31 (22.0)^a^	20 (10.6)^b^	<0.001^∗^
1.3 ≥ PIR < 3.5	743 (36.9)	229 (35.1)	62 (36.1)	82 (39.2)	0.703	215 (33.5)	63 (38.8)	92 (49.5)^b^	0.029^∗^
PIR <1.3	1011 (33.7)	288 (30.6)	85 (38.8)	117 (33.9)	0.235	301 (32.0)	94 (39.2)	126 (39.9)	0.172
Perceived weight status, *n* (%)									
Thin	161 (5.7)	97 (11.2)	3 (0.9)^a^	3 (0.8)^b^	<0.001^∗^	54 (6.0)	2 (0.4)^a^	2 (1.0)^b^	<0.001^∗^
Normal	1680 (71.0)	638 (86.7)	160 (78.0)^a^	88 (32.9)^bc^	<0.001^∗^	636 (87.2)	101 (50.9)^a^	57 (23.6)^bc^	<0.001^∗^
Overweight/fat	597 (23.3)	22 (2.1)	47 (21.0)^a^	167 (66.3)^bc^	<0.001^∗^	59 (6.8)	97 (48.6)^a^	205 (75.5)^bc^	<0.001^∗^
Physical activity									
Meet physical activity recommendation^$^, *n* (%)	1354 (62.8)	525 (75.8)	141 (73.5)	159 (66.0)^b^	0.048^∗^	342 (57.6)	88 (46.2)	99 (38.8)^b^	0.045^∗^
Physical activity (minutes/day)	81.1 ± 2.5	101.0 ± 5.1	108.3 ± 9.0	97.1 ± 12.0	0.592	64.1 ± 3.9	63.3 ± 10.7	46.0 ± 4.7^b^	0.023^∗^
Reported work-related physical activity, *n* (%)	808 (39.0)	282 (41.4)	85 (51.8)^a^	85 (41.5)^c^	0.042^∗^	205 (33.6)	71 (42.1)^a^	80 (32.4)^c^	0.010^∗^
Reported travel-related physical activity, *n* (%)	1125 (42.2)	377 (46.3)	102 (45.7)	143 (51.8)	0.628	302 (35.4)	82 (39.7)	119 (39.3)	0.899
Reported recreation-related physical activity, *n* (%)	1817 (78.8)	638 (85.7)	174 (84.2)	198 (78.4)	0.088	508 (76.5)	128 (68.2)	171 (68.6)	0.783
Sedentary behaviors									
Sitting time (minutes/day)	492.7 ± 7.6	465.3 ± 8.6	472.8 ± 18.4	522.8 ± 18.0^bc^	0.002^∗^	505.1 ± 10.3	463.3 ± 19.8^a^	543.2 ± 13.1^bc^	0.002^∗^
Screen time (hours/day)	3.6 ± 0.1	3.6 ± 0.1	3.8 ± 0.2	4.3 ± 0.2^bc^	0.037^∗^	3.4 ± 0.1	3.4 ± 0.2	4.0 ± 0.2^bc^	<0.001^∗^
Screen time ≤2 hours/day, *n* (%)	844 (37.3)	248 (35.7)	76 (36.1)	78 (27.8)	0.188	292 (43.0)	67 (40.4)	83 (33.4)^b^	0.058
Weight loss intention, *n* (%)^#^	509 (38.1)	53 (13.3)	55 (46.4)^a^	116 (75.8)^bc^	<0.001	93 (22.8)	68 (73.5)^a^	124 (82.8)^b^	<0.001^∗^

*Note*. NHANES = National Health and Nutrition Examination Survey; MET = metabolic equivalent scores; PIR = family income to poverty ratio; sample weight to conduct all the analyses; data are presented as mean ± SE except otherwise specified; ^$^*p* value was obtained performing SURVEYREG; ^&^*p* values were obtained by performing SURVEYFREQ (CHISQ, based on the Rao–Scott chi-square test with an adjusted *F* statistic); all the other *p* values for continuous variable were obtained by performing SURVEYREG, and for category variables, values were obtained by performing SURVEYLOGISTIC to perform the adjusted analyses, adjusted for age, race, parental education level, and PIR; ^$^accumulated ≥1680 MET-minutes/week for 12–17 years old, and accumulated ≥600 MET-minutes/week for 18–19 years old; ^#^data were only available for participants aged 12–15 years; ^a^normal weight differs from overweight (*p* < 0.05); ^b^normal weight differs from obese (*p* < 0.05); ^c^overweight differs from obese (*p* < 0.05).

**Table 2:** Weight perception and health behaviors in adolescents aged 12–19 years, NHANES 2011–2014.

	Total	Thin	Normal	Overweight/fat	Overall *p* value
*Boys*					
12–19 years	*n*=1225	*n*=103 (7.3%)	*n*=886 (74.1%)	*n*=236 (18.6%)	
Physical activity					
Meet physical activity recommendation^$^, *n* (%)	825 (73.4)	70 (67.2)	616 (76.3)	139 (64.0)^a^	<0.001^∗^
Physical activity (minutes/day)	101.4 ± 4.6	117.3 ± 19.1	101.2 ± 4.4	96.3 ± 10.8	0.187
Sedentary behaviors					
Sitting time (minutes/day)	478.5 ± 8.0	436.0 ± 20.1	474.1 ± 10.2	512.7 ± 16.3^ab^	<0.001^∗^
Screen time (hours/day)	3.7 ± 0.1	3.6 ± 0.3	3.6 ± 0.1	4.4 ± 0.2^ab^	0.004^∗^
Screen time ≤2 hours/day, *n* (%)	402 (34.1)	33 (31.4)	299 (36.4)	70 (26.0)^a^	0.017^∗^
12–15 years	*n*=626	*n*=49 (6.2%)	*n*=465 (78.1%)	*n*=112 (15.8%)	
Weight loss intention, *n* (%)^#^	224 (32.0)	3 (12.1)	119 (22.4)	102 (87.5)^ab^	<0.001
*Girls*					
12–19 years	*n*=1213	*n*=58 (4.1%)	*n*=794 (67.9%)	*n*=361 (28%)	
Physical activity					
Meet physical activity recommendation^$^, *n* (%)	529 (51.8)	22 (46.1)	369 (56.6)	138 (41.0)^a^	0.008^∗^
Physical activity (minutes/day)	60.0 ± 2.6	61.1 ± 11.4	64.6 ± 3.3	48.7 ± 5.0^a^	0.021^∗^
Sedentary behaviors					
Sitting time (minutes/day)	507.3 ± 9.0	489.8 ± 23.4	508.6 ± 10.6	506.7 ± 15.1	0.377
Screen time (hours/day)	3.5 ± 0.1	3.1 ± 0.4	3.5 ± 0.1	3.8 ± 0.2	0.135
Screen time ≤2 hours/day, *n* (%)	442 (40.5)	23 (44.7)	311 (43.2)	108 (33.3)	0.153
12–15 years	*n*=608	*n*=32 (4.7%)	*n*=423 (69.9%)	*n*=153 (25.3%)	
Weight loss intention, *n* (%)^#^	285 (44.3)	3 (9.8)	143 (29.4)^c^	139 (91.6)^ab^	<0.001

*Note*. NHANES = National Health and Nutrition Examination Survey; MET = metabolic equivalent scores; sample weight to conduct all the analyses; data are presented as mean ± SE or otherwise specified; the other *p* values for continuous variable were obtained by performing SURVEYREG, and *p* values for category variables were obtained by performing SURVEYLOGISTIC to perform the adjusted analyses, adjusted for age, race, parental education level, and family income to poverty ratio; ^$^accumulated ≥1680 MET-minutes/week for 12–17 years old, and accumulated ≥600 MET-minutes/week for 18–19 years old; ^#^data were only available for participants aged 12–15 years; ^a^normal differs from overweight (*p* < 0.05); ^b^thin differs from overweight (*p* < 0.05); ^c^thin differs from normal weight (*p* < 0.05).

**Table 3:** Weight perception accuracy and health behaviors in adolescents aged 12–19 years, NHANES 2011–2014.

	Normal weight			Overweight			Obese		
	Accurate	Inaccurate	*p* value	Accurate	Inaccurate	*p* value	Accurate	Inaccurate	*p* value
*Boys*									
12–19 years	*n*=638 (87%)	*n*=119 (13%)		*n*=47 (21%)	*n*=163 (79%)		*n*=167 (66%)	*n*=91 (34%)	
Physical activity									
Meet physical activity recommendation^$^, *n* (%)	448 (77.4)	77 (64.8)	0.244	29 (71.1)	112 (74.2)	0.403	97 (62.4)^a^	62 (72.9)	0.102
Physical activity (minutes/day)	100.1 ± 4.8	107.0 ± 17.6	0.686	108.4 ± 22.5	108.2 ± 10.0	0.703	96.7 ± 14.7	98.0 ± 18.3	0.271
Sedentary behaviors									
Sitting time (minutes/day)	468.1 ± 9.6	447.0 ± 19.2	0.839	430.8 ± 41.6	483.8 ± 18.9	0.502	535.6 ± 21.3^ab^	497.8 ± 31.9	0.007^∗^
Screen time (hours/day)	3.5 ± 0.1	3.7 ± 0.3	0.893	4.5 ± 0.5	3.6 ± 0.2	0.309	4.4 ± 0.3^a^	3.9 ± 0.3	0.137
Screen time ≤2 hours/day, *n* (%)	210 (36.4)	38 (30.8)	0.705	14 (29.7)	62 (37.8)	0.84	49 (24.7)^a^	29 (34.0)	0.036^∗^
12–15 years	*n*=329 (89%)	*n*=55 (11%)		*n*=22 (17%)	*n*=82 (83%)		*n*=81 (57%)	*n*=57 (43%)	
Weight loss intention, *n* (%)^#^	46 (12.8)	7 (17.4)	0.494	19 (80.3)^c^	36 (39.4)	0.046^∗^	77 (90.3)^a^	39 (56.4)	0.004^∗^
*Girls*									
12–19 years	*n*=636 (87%)	*n*=113 (13%)		*n*=97 (49%)	*n*=103 (51%)		*n*=205 (76%)	*n*=59 (24%)	
Physical activity									
Meet physical activity recommendation^$^, *n* (%)	300 (58.8)	42 (49.0)	0.276	39 (43.5)	49 (48.7)	0.185	78 (37.4)^a^	21 (43.2)	0.251
Physical activity (minutes/day)	64.2 ± 3.5	63.3 ± 12.3	0.919	57.8 ± 12.8	68.6 ± 15.4	0.351	41.0 ± 4.1^a^	61.7 ± 12.2	0.005^∗^
Sedentary behaviors									
Sitting time (minutes/day)	507.9 ± 11.7	486.3 ± 15.4	0.192	463.5 ± 26.7	463.2 ± 23.7	0.329	533.3 ± 15.9^b^	573.0 ± 22.8	0.227
Screen time (hours/day)	3.4 ± 0.1	3.3 ± 0.2	0.847	3.5 ± 0.2	3.3 ± 0.2	0.928	4.1 ± 0.2^ab^	4.0 ± 0.5	0.716
Screen time ≤2 hours/day, *n* (%)	249 (42.9)	43 (43.3)	0.585	29 (35.8)	38 (44.9)	0.208	57 (29.7)^a^	26 (45.0)	0.263
12–15 years	*n*=315 (87%)	*n*=53 (13%)		*n*=29 (37%)	*n*=67 (63%)		*n*=102 (70%)	*n*=42 (30%)	
Weight loss intention, *n* (%)^#^	71 (19.8)	22 (43.1)	0.015^∗^	28 (96.8)^c^	40 (59.9)	0.002^∗^	91 (89.5)^a^	33 (66.9)	0.046^∗^

*Note*. NHANES = National Health and Nutrition Examination Survey; MET = metabolic equivalent scores; sample weight to conduct all the analyses; data are presented as mean ± SE or otherwise specified; the other *p* values for continuous variable were obtained by performing SURVEYREG, and *p* values for category variables were obtained by performing SURVEYLOGISTIC to perform the adjusted analyses, adjusted for age, race, parental education level, and family income to poverty ratio; ^$^accumulated ≥1680 MET-minutes/week for 12–17 years old, and accumulated ≥600 MET-minutes/week for 18–19 years old; ^#^data were only available for participants aged 12–15 years; ^a^normal differs from obese (*p* < 0.05); ^b^overweight differs from obese (*p* < 0.05); ^c^normal differs from overweight (*p* < 0.05).
